# Mapping the global geographic potential of Zika virus spread

**DOI:** 10.1590/0074-02760160149

**Published:** 2016-09

**Authors:** Abdallah M. Samy, Stephanie M Thomas, Ahmed Abd El Wahed, Kevin P Cohoon, A. Townsend Peterson

**Affiliations:** 1 Ain Shams University, Faculty of Science, Cairo, Egypt; 2 University of Kansas, Biodiversity Institute, Lawrence, KS, USA; 3 University of Bayreuth, Department of Biogeography, Bayreuth, Germany; 4 Georg-August University, Division of Microbiology and Animal Hygiene, Goettingen, Germany; 5 Mayo Clinic, Rochester, MN, USA

**Keywords:** Zika virus, global distribution, risk, Aedes, Brazil, climate, socioeconomic, accessibility

## Abstract

The Americas are presently experiencing the most serious known outbreak of Zika virus (ZIKV). Here, we present a novel set of analyses using environmental characteristics, vector mosquito distributions, and socioeconomic risk factors to develop the first map to detail global ZIKV transmission risk in multiple dimensions based on ecological niche models. Our model predictions were tested against independent evaluation data sets, and all models had predictive ability significantly better than random expectations. The study addresses urgent knowledge gaps regarding (1) the potential geographic scope of the current ZIKV epidemic, (2) the global potential for spread of ZIKV, and (3) drivers of ZIKV transmission. Our analysis of potential drivers of ZIKV distributions globally identified areas vulnerable in terms of some drivers, but not for others. The results of these analyses can guide regional education and preparedness efforts, such that medical personnel will be better prepared for diagnosis of potential ZIKV cases as they appear.

Zika virus (ZIKV) is a member of the family *Flaviviridae*, transmitted to humans via bites of infected *Aedes* (*Ae. aegypti* and *Ae. albopictus*) mosquitoes. ZIKV is spreading rapidly in the Americas; indeed, the World Health Organization anticipates 4 million cases in 2016 (Koenig 2016). ZIKV disease is usually a mild febrile illness with rash and conjunctivitis, but global concern about ZIKV transmission centres on increased incidence of microcephaly and other birth defects in fetuses born to mothers infected with ZIKV (Melo et al. 2016, Mlakar et al. 2016, Ventura et al. 2016). Guillain-Barré syndrome has also co-occurred with ZIKV emergence in the Americas, as it did previously in French Polynesia (Oehler et al. 2014). ZIKV’s geographic potential is not well understood, emphasising the need for models that consider the entire transmission cycle as recent models (Bogoch et al. 2016, Monaghan et al. 2016) have considered only vector distribution and human travel in the Americas.

We used maximum entropy ecological niche modeling implemented in Maxent version 3.3 (Phillips et al. 2006) to assess and anticipate the potential distribution of ZIKV worldwide, and to infer major drivers of the virus’ spread. We developed four models, based on ZIKV occurrences and different combinations of climate, socioeconomic, land-cover, mosquito abundance, and accessibility variables (see Supplementary data for details of data sources and methods). Models were calibrated across Mexico, Central, and South America, and then projected worldwide for interpretation. For each combination of drivers, we ran 100 bootstrap replicates; the median of those replicates was used as an estimate for the ZIKV ecological niche. These models were thresholded based on a maximum allowable omission error rate of 5% [*E* = 5%; (Peterson et al. 2008)]. For visualisation, we combined thresholded versions of two of these models to illustrate differences in prediction deriving from different combinations of possible drivers of ZIKV transmission. Model predictions were evaluated using partial receiver operating characteristic (ROC) tests applied to random subsets of 50% of available occurrence data (Peterson et al. 2008).

Our results constitute the first detailed, multiple-driver predictions of ZIKV potential distribution worldwide that also allow assessing and identifying possible drivers of risk (see Figure and Supplementary data). All model predictions had predictive ability regarding independent subsets of occurrence data significantly better than random expectations (all p < 0.001). Our models corroborated ZIKV’s large-scale potential for expansion in South and Central America, and identified other regions at risk of transmission, particularly in Sub-Saharan Africa, Australia, Melanesia, and parts of New Zealand. Northern Australia was at risk as a function of vector availability and environmental suitability, but less so based on human conditions. Other at-risk regions included Angola, Zambia, the Amazon basin, and northern South America. Risk in Europe and northern India appeared driven by accessibility and socioeconomic factors, respectively.

Our models anticipated some potential for autochthonous ZIKV transmission in the USA, although areas identified were generally scattered and narrow: Florida, southern Texas, and Louisiana are clearly vulnerable to autochthonous ZIKV transmission. Additional areas southeast of the Appalachians and in Pacific coastal areas may also be affected. Other parts of the USA will see imported cases and limited local transmission, particularly if mosquito species other than *Ae. aegypti* and *Ae. albopictus* participate in transmission. Large areas of Asia, including densely populated regions, were indicated as at risk for autochthonous ZIKV transmission, including parts of India, Bangladesh, southern China, and Thailand; the southwestern coast of the Arabian Peninsula is also at risk. In Western Europe, ZIKV transmission risk is enhanced by travel times and connectivity to known transmission areas; as such, isolated autochthonous cases may occur, at least seasonally, when competent vector species are present.

**Figure:**
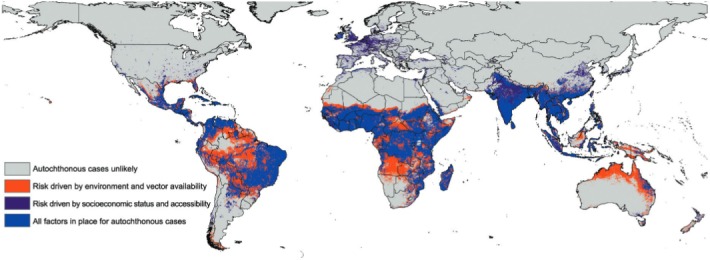
Predicted global potential distribution of Zika virus, based on ecological niche models integrating occurrences with data on climate, socioeconomic status, land-cover, mosquito abundance, and accessibility. Orange areas were identified as suitable based on drivers related to physical environment and vector populations; purple areas were identified as suitable based on drivers related to human conditions and accessibility; blue areas were identified as suitable in terms of all drivers considered (individual models are presented in the Supplementary data). Note some potential for autochthonous transmission in the southeastern USA, but broader potential for accessibility-related cases (*e.g.*, imported infections that may turn into autochthonous transmission via seasonal vector activity) across the USA and Europe. A raster GIS (5 km resolution) version of this map is available from: https://figshare.com/s/0257ff447ccc11373e41.

Our model adds key parameters to the present picture of risk of ZIKV arrival, establishment, and autochthonous transmission worldwide, for a more comprehensive model than has been available to date. Healthcare providers and health authorities in areas of ZIKV transmission risk should be on alert for infected individuals, and health authorities should advise healthcare providers of the risk, and residents, especially pregnant women, of the need for use of anti-vector measures. Our set of models identified areas at risk, and predicted successfully the recent and historic ZIKV outbreaks in both local and global scale; however, they does not show detailed transmission on finer resolutions, such that individual cases may appear via other routes of transmission (*e.g.*, sexual transmission). ZIKV transmission risk and disease can be reduced by (1) reduction of mosquito-human contact by reducing mosquito populations and eliminating breeding sites; (2) enhanced public and clinical awareness of ZIKV risk; (3) prompt reporting of new cases to public health authorities; (4) research on the ecology, evolution, clinical manifestations, vector associations, and transmission dynamics of ZIKV; and (5) prospective screening for potential cases in areas at high risk. A crucial question in anticipating a next generation of such models is the role of vector mosquito species beyond the globally distributed *Ae. aegypti* and *Ae. albopictus*.
