# Supplementary material for: Mapping the global geographic potential of Zika virus spread
Source: Mem Inst Oswaldo Cruz. 2016 Sep;111(9):559–60. doi: 10.1590/0074-02760160149 (PMC5027865; doi:10.1590/0074-02760160149)
Supplement: Supplementary file 1 [file 0074-0276-mioc-0074-02760160149-suppl01.pdf]

MODELING METHODS

Occurrences of Zika virus - Records of the occurrence of Zika virus (ZIKV) were compiled from ProMED-mail (http://www.promedmail.org) and HealthMap (http://www.healthmap.org), and the Brazilian Ministry of Health (http://portalsaude.saude.gov.br/images/pdf/2015/novembro/30/Microcefalia-2-boletim.pdf). Records were filtered to remove duplicate coordinates, and inspected to remove any cases that had been corrected or retracted. The data set for model calibration includes records from Mexico, Central America, and South America, as only these regions had data sufficiently dense for rigorous model calibration. We randomly divided these records into two equal sets: one to be used for model calibration and the other for model evaluation.

Model covariates - Several suites of environmental variables were used as independent variables in our models to characterise environmental variation across the calibration area and globally. These variables were considered as potential drivers of both direct and indirect effects on emergence of mosquito-borne diseases (Peterson 2014). These variables include daytime and nighttime land surface temperature and enhanced vegetation index (EVI) values for January 2012-July 2015 derived from Moderate Resolution Imaging Spectroradiometer (MODIS) satellite imagery (source: http://reverb.echo.nasa.gov/reverb/#utf8=%E2%9C%93&spatial_map=satellite&spatial_type=rectangle). EVI approximates photosynthetic mass (termed colloquially 'greenness'), and offers a proxy measure of soil moisture, an important factor in determining larval mosquito habitats (Estallo et al. 2008, Nihei et al. 2014).

Precipitation, aridity and water stress are also important potential factors in the distribution of ZIKV, in light of their major roles in determining mosquito breeding sites. Effects of precipitation, aridity and soil water stress on breeding habitats for ZIKV vector species (*Aedes* spp.) have been previously examined (Kalra et al. 1997, Alto & Juliano 2001, Wu et al. 2009, Mogi et al. 2015). An important result is that drought drives human need for water storage in containers, leading to elevated mosquito populations and increased biting rates (Kalra et al. 1997); indeed, Brazil has faced recent drought, which increased water storage (Sena et al. 2014). To account for these factors, we used data for (1) maximum and minimum monthly precipitation data from WorldClim (http://www.worldclim.org) and (2) aridity and soil water stress data layers from the Consortium for Spatial Information (CGIAR-CSI; http://www.cgiar-csi.org/data/global-aridity-and-pet-database), respectively. For soil water stress, we calculated composite indices as maxima and minima across all of the monthly estimates.

Land cover has been identified as key in breeding habitats of *Aedes* spp., the likely prime vectors of ZIKV (Vanwambeke et al. 2007). We thus used a global land cover layer available from WorldGrids (http://worldgrids.org/doku.php?id=wiki:layers#land_cover_and_land_use). We accounted for elevational gradients identified as influential factors in disease dynamics and mosquito abundance by means of a layer summarising elevation available from the Shuttle Radar Topography Mission (SRTM; http://srtm.usgs.gov/) (Lozano-Fuentes et al. 2012, Dhimal et al. 2015).

Disease transmission and spread dynamics, however, are not dependent solely on environmental factors; other sets of variables also play major roles (Koyadun et al. 2012, Teurlai et al. 2015). Hence, we used grids of human population density, nighttime lights, and accessibility via transportation. Population density grids were drawn from the Gridded Population of the World, version 4 (GPWv4), collection released recently via http://beta.sedac.ciesin.columbia.edu/data/collection/gpw-v4. Nighttime satellite imagery (year 2013) was used as a proxy for real poverty (Noor et al. 2008, Wang et al. 2012), and was obtained from NOAA-Defense Meteorological Satellite Program (http://ngdc.noaa.gov/eog/dmsp/downloadV4composites.html). Accessibility was summarised in terms of travel time by land or sea (Nelson 2008), as the connectivity between population sites is an important variable in potential distributions of emerging diseases (Cliff & Haggett 2004); this layer was developed by the European Commission and World Bank (http://forobs.jrc.ec.europa.eu/products/gam/download.php). Finally, we used two grids representing the environmental suitability for *Ae. aegypti* and *Ae. albopictus* as potential vectors for ZIKV derived from a recent detailed analysis (Kraemer et al. 2015), available from: http://goo.gl/Zl2P7J; and developed parallel models based on another recent analysis of the same two species (Campbell et al. 2015). These mosquito variables have been summarised in raster format at a spatial resolution of 5 km, the same resolution used in our study, and have been used in a previous study of similar purposes (Nsoesie et al. 2016). All grids were resampled to a spatial resolution of 5 x 5 km in ArcMap 10.3, in light of the global distribution of the species.

Ecological niche modeling - We approximated the ZIKV fundamental ecological niche via ecological niche modeling (ENM) via the maximum entropy algorithm implemented in Maxent, version 3.3 (Phillips et al. 2006). Peterson et al. (2011) defined the fundamental niche as "the set of environmental conditions required for the species to maintain populations without immigrational subsidy". ENM relates known occurrences of species to the set of environmental variables in a maximum entropy, evolutionary-computing environment to approximate this set of environmental conditions associated with maintenance of populations (Peterson et al. 2011).

We calibrated ENMs across Mexico, Central America and South America, where ZIKV occurrence data were sufficiently dense for rigorous model calibration (Owens et al. 2013); models were then projected worldwide for interpretation. To explore and understand contributions of different suites of variables to shaping the distribution of ZIKV, we used different combinations of environmental variables; socioeconomic variables and accessibility (see Neerinckx et al. 2008). These explorations illuminate the roles of possible drivers of ZIKV transmission beyond just climate (Kilpatrick & Randolph 2012, Weaver 2013). A full elaboration of combinations of drivers that we explored is presented in Supplementary Table.

SUPPLEMENTARY TABLE

Combinations of environmental variables used to calibrate the ecological niche models for ZIKV in this study

Variable	Model 1	Model 2	Model 3	Model 4
Population density 2015	X	√	X	√
Daytime temperature	√	√	√	√
Nighttime temperature	√	√	√	√
Maximum monthly precipitation	√	√	√	√
Minimum monthly precipitation	√	√	√	√
Enhanced vegetation index	√	√	√	√
Maximum soil water stress	√	√	√	√
Minimum soil water stress	√	√	√	√
Aridity	√	√	√	√
Elevation	√	√	√	√
Land-cover	X	√	X	√
Aedes aegypti abundance	X	X	√	√
Aedes albopictus abundance	X	X	√	√
Nighttime lights	X	√	X	√
Accessibility	X	√	X	√

Check marks indicate that the variable was used in the model; X's: indicate variables that were not used in the model.

For each combination, we ran Maxent using 100 bootstrap replicates. The median of the outputs of the replicates was used as a best final estimate in subsequent analyses. Final models were thresholded based on a maximum allowable omission error rate of 5% [E = 5%; (Peterson et al. 2008)], in effect assuming that ≤ 5% of occurrence data would have sufficient error in geolocation that variable values might be misrepresented.

Model predictions were evaluated for statistical significance based on predictions among random subsets of 50% of available data. In effect, these tests assess whether a model based on a certain amount of occurrence information across a region will be able to anticipate the next set of occurrences across that same landscape; while not an ideal test of predictive ability when models are transferred to other regions, it is at present the only test available to us. We used partial receiver operating characteristic (ROC) statistics (Peterson et al. 2008), which avoid well-known problems with traditional ROC approaches (Lobo et al. 2008). Partial ROC statistics were calculated using the PartialROC function in the ENMGadgets package in R software version 3.2.0 (R Development Core Team 2015), specifying the same E = 5%, a 50% bootstrap resampling, and 100 random iterations (Lobo et al. 2008).

For visualisation, we combined two of these thresholded models (Model 3 and Model 4) to illustrate differences between predictions based on different possible drivers of ZIKV transmission. Interesting contrasts emerge from differences in suitability based on all of the environmental dimensions, areas identified as suitable based on climate and presence of vector species, and suitability in terms of human socioeconomic variables and accessibility, as is exemplified in the Figure in the main paper. GIS-readable grids (GeoTIFF format) and Google Earth keyhole markup language (KML) files are available via Figshare (https://figshare.com/s/0257ff447ccc11373e41).

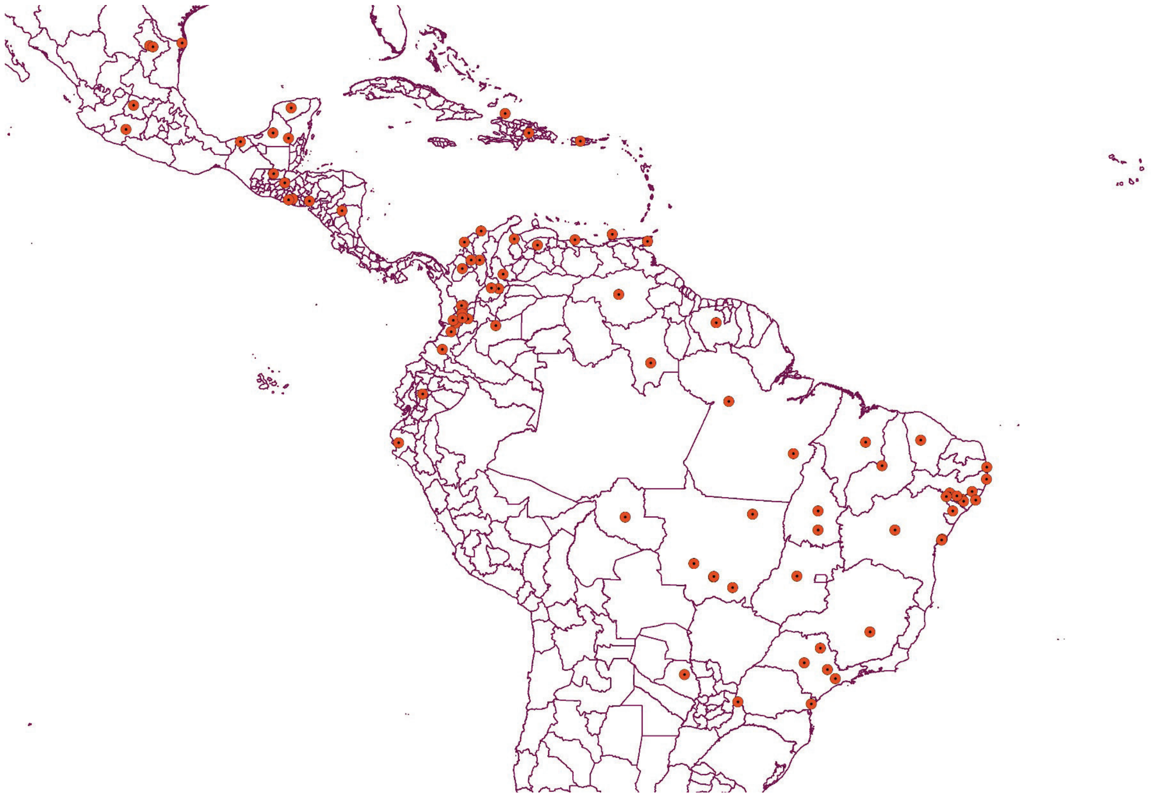

Supplementary figure 1: ZIKV occurrence records (dotted circles) used in model calibration across Mexico, Central America, and South America.

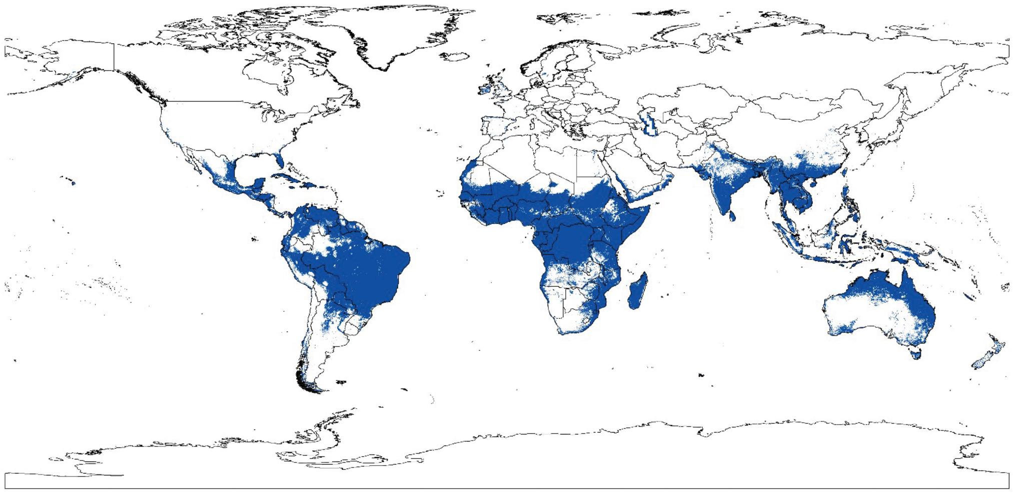

Supplementary figure 2: potential geographic distribution of ZIKV (in blue) based on analysis of only environmental variables (Model 1), based on a threshold that admits a maximum of 5% omission error.

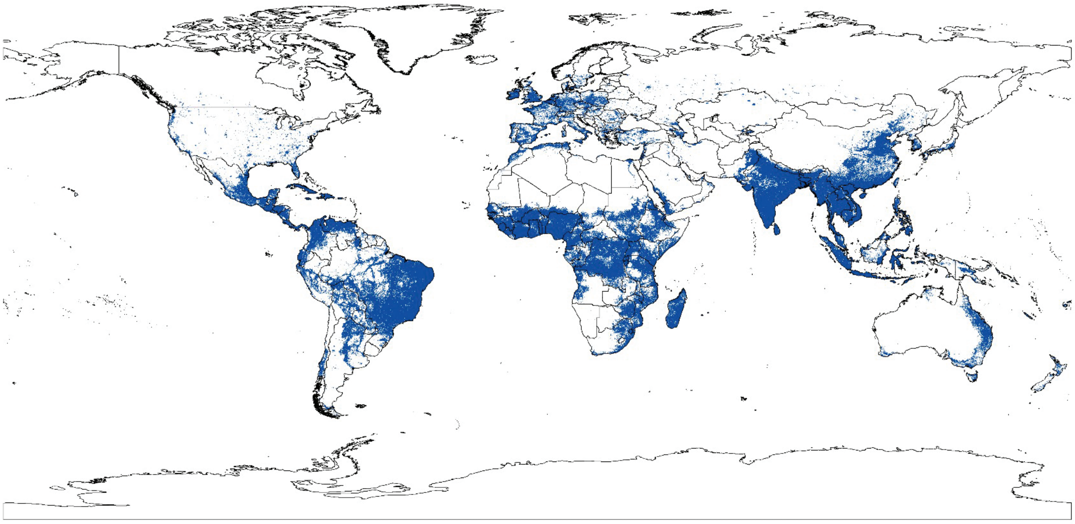

Supplementary figure 3: potential geographic distribution of ZIKV (in blue) based on analysis of all environmental, socioeconomic, poverty proxy and accessibility variables (Model 2), based on a threshold that admits a maximum of 5% omission error.

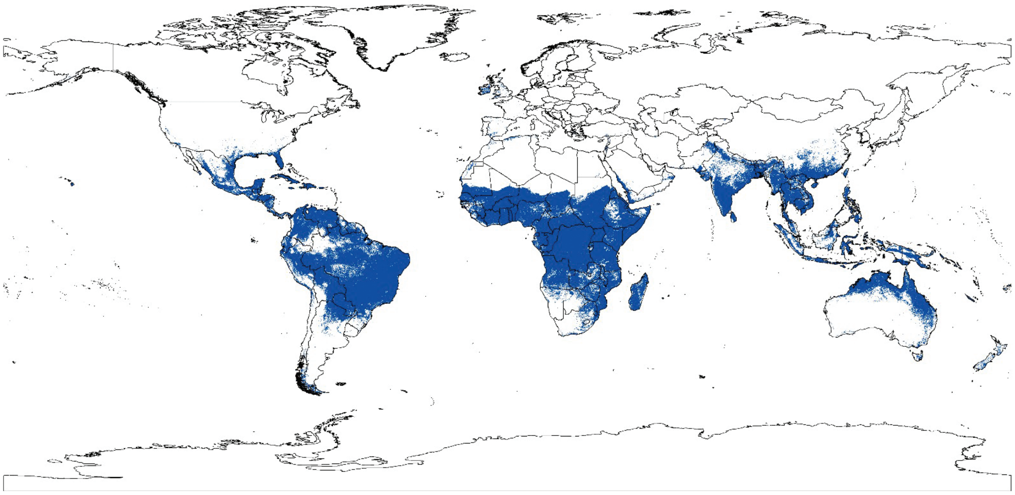

Supplementary figure 4: potential geographic distribution of ZIKV (in blue) based on analysis of environmental variables and mosquito density (Model 3), based on a threshold that admits a maximum of 5% omission error.

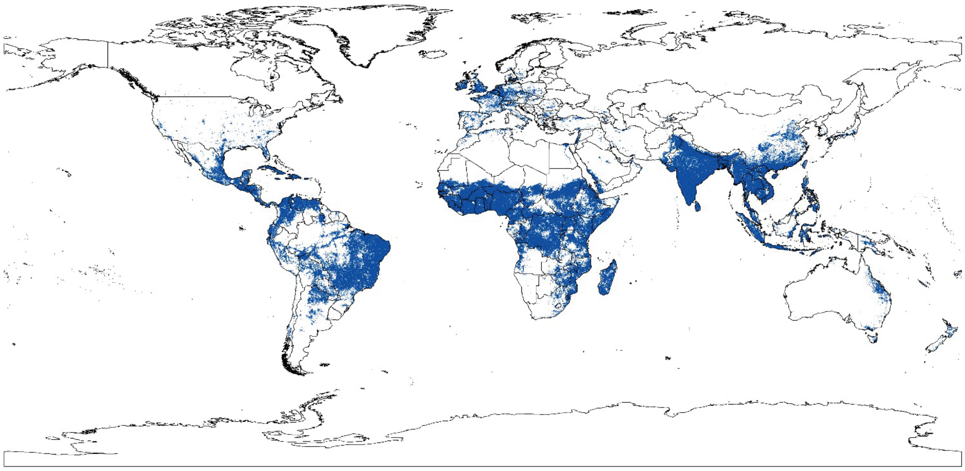

Supplementary figure 5: potential geographic distribution of ZIKV (in blue) based on analysis of all environmental, socioeconomic, mosquito-related, poverty-related and accessibility variables (Model 4), based on a threshold that admits a maximum of 5% omission error.

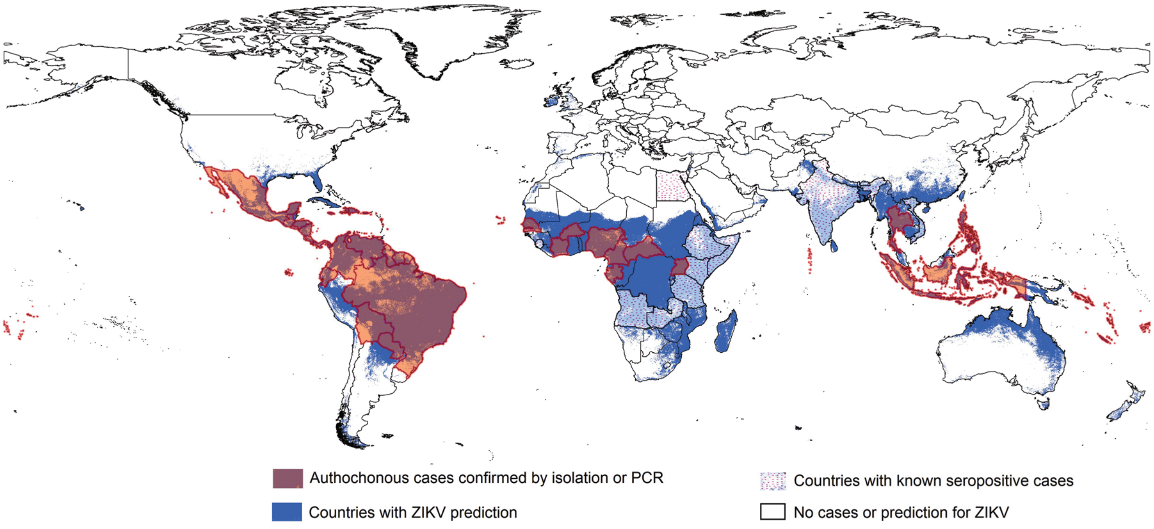

Supplementary figure 6: potential geographic distribution of ZIKV based on environmental variables only (Model 1) in relation to known global distribution of the virus. Note that models were calibrated based on occurrences in Mexico, Central America, and South America, such that occurrences in Africa, Asia, and the Pacific had no contribution to model calibration. Countries reported with autochthonous cases confirmed by isolation or PCR (orange shading with brown boundaries) and countries with known seropositive cases (red stippled areas and light-blue shading) are shown in relation to the model predictions (in blue, and purple and light blue where overlapped by the known-positive cases. Note the generally close correspondence between the global projection of our models and countries where ZIKV has been detected via virus isolation, PCR, or serological studies.

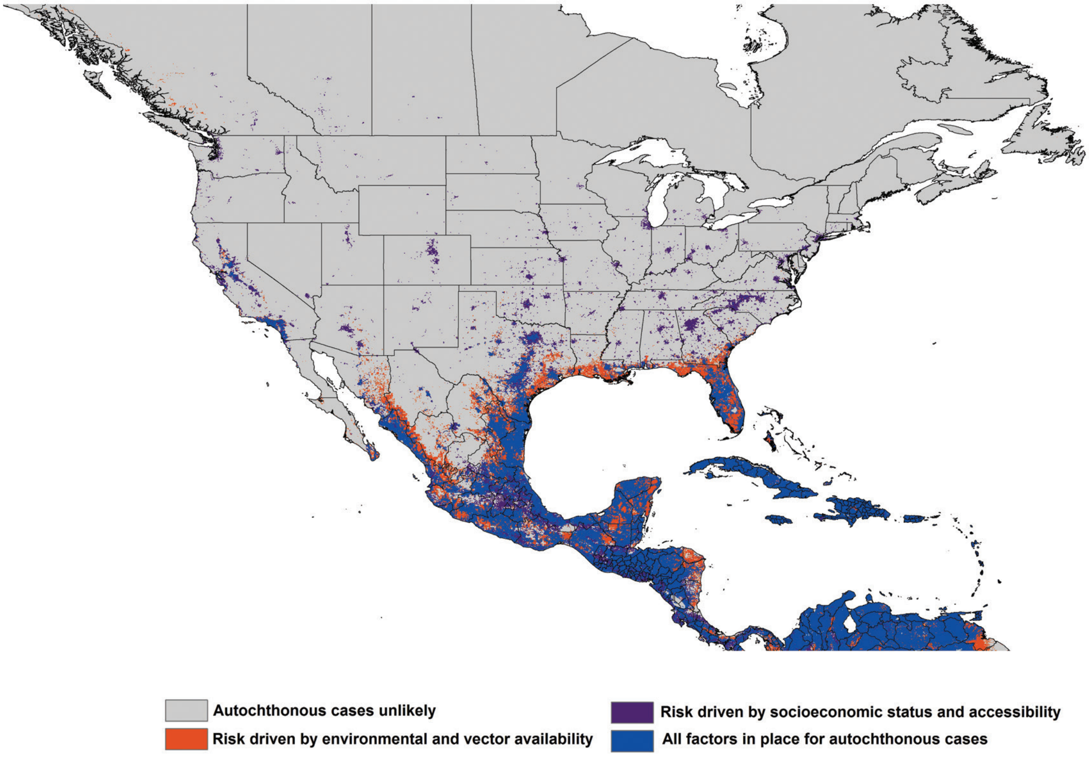

Supplementary figure 7: close-up of North America to provide detail additional to the figure in the main paper. Orange areas were identified as suitable based on drivers related to physical environment and vector populations; purple areas were identified as suitable based on drivers related to human conditions and accessibility; blue areas were identified as suitable in terms of all drivers considered.

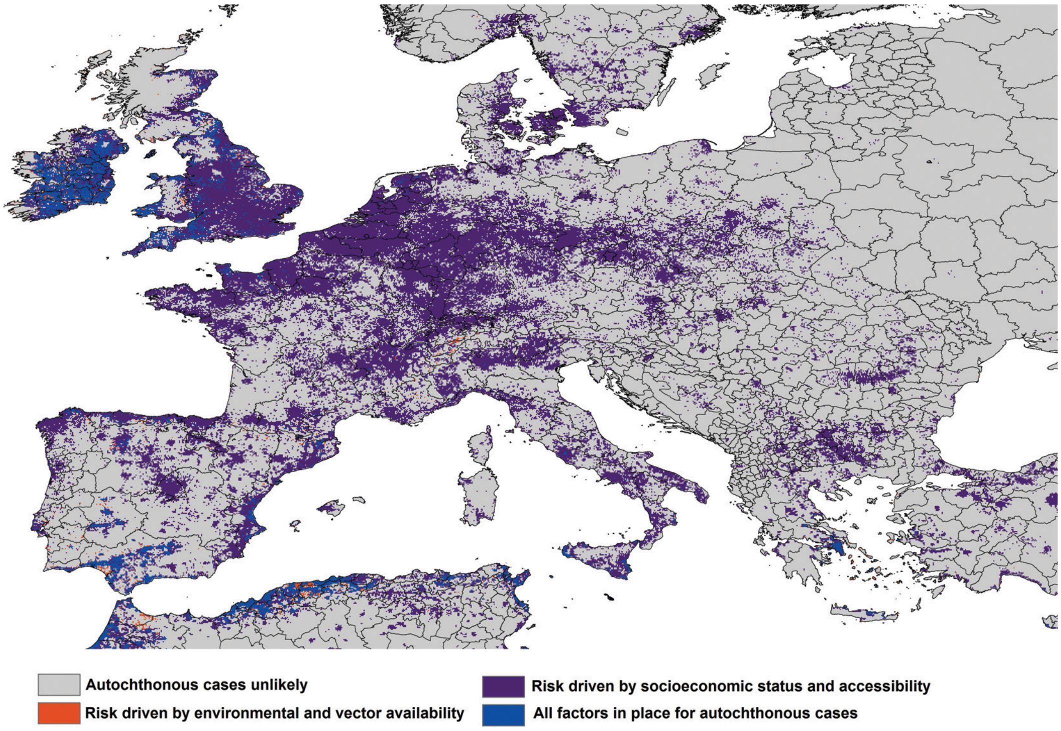

Supplementary figure 8: close-up of Europe to provide detail additional to the figure in the main paper. Orange areas were identified as suitable based on drivers related to physical environment and vector populations; purple areas were identified as suitable based on drivers related to human conditions and accessibility; blue areas were identified as suitable in terms of all drivers considered.

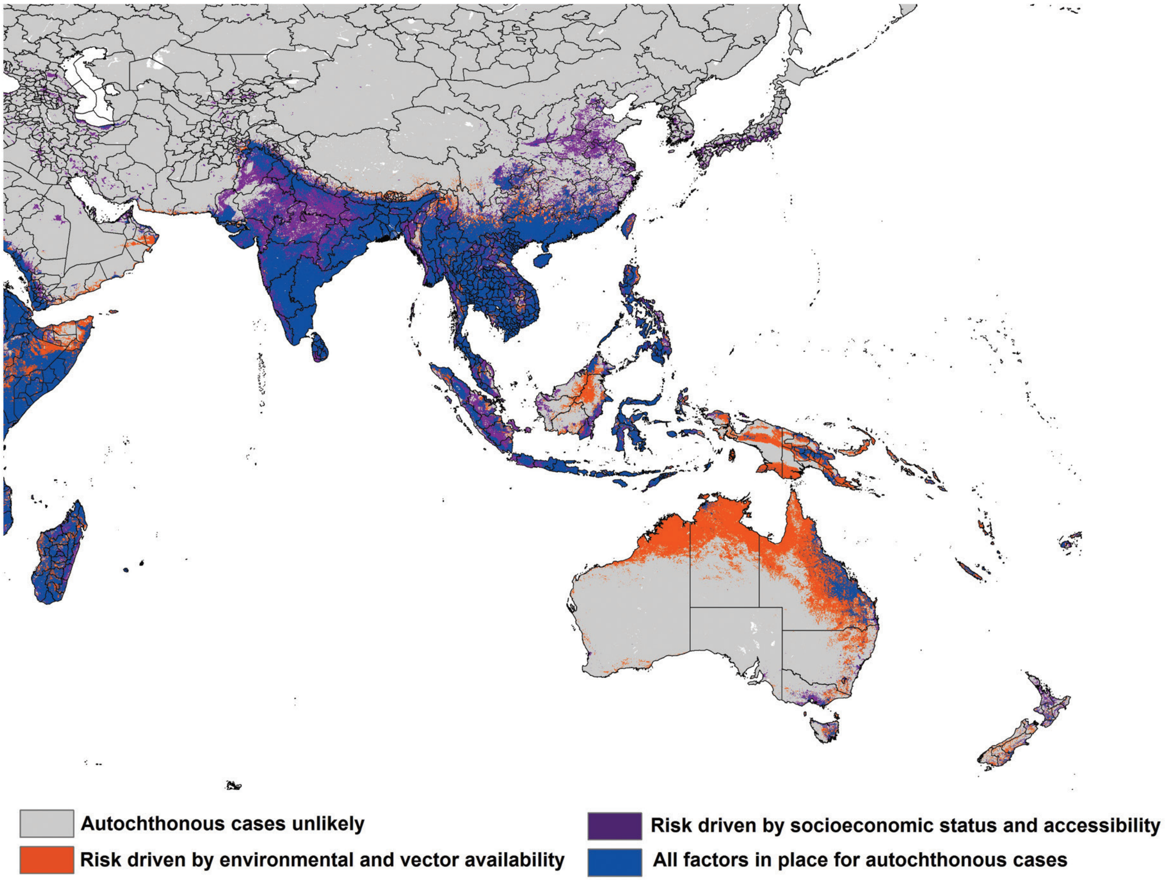

Supplementary figure 9: close-up of Asia and Australia to provide detail additional to the figure in the main paper. Orange areas were identified as suitable based on drivers related to physical environment and vector populations; purple areas were identified as suitable based on drivers related to human conditions and accessibility; blue areas were identified as suitable in terms of all drivers considered.

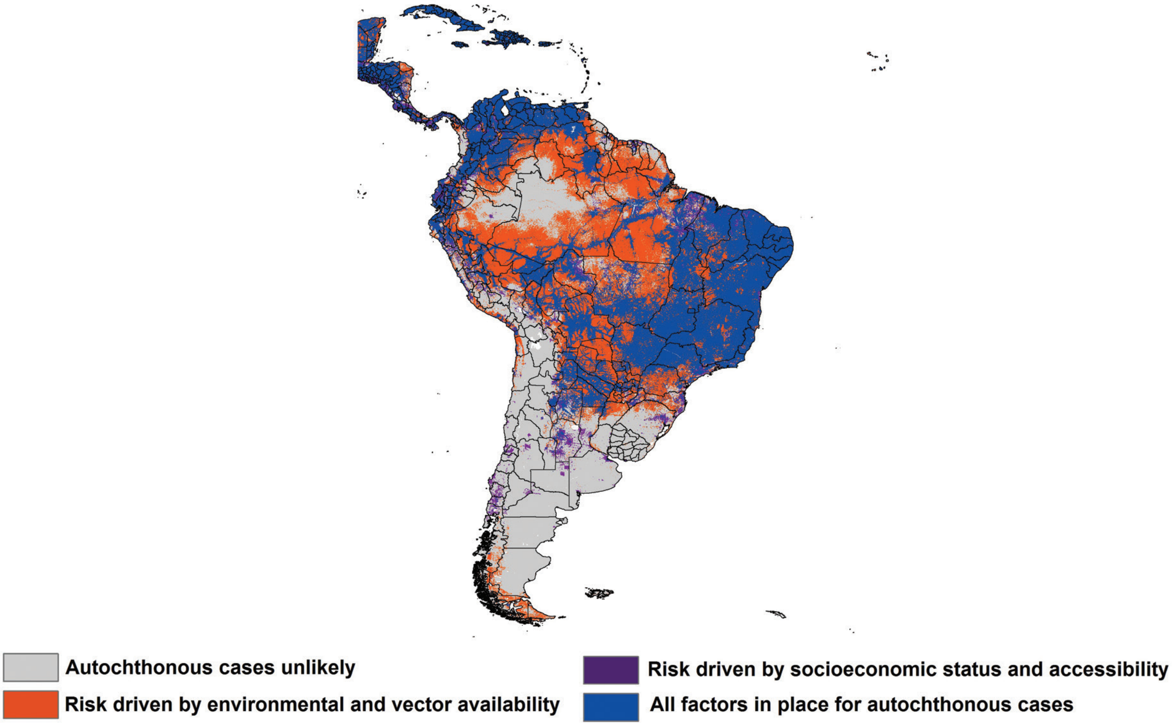

Supplementary figure 10: close-up of South America to provide detail additional to the figure in the main paper. Orange areas were identified as suitable based on drivers related to physical environment and vector populations; purple areas were identified as suitable based on drivers related to human conditions and accessibility; blue areas were identified as suitable in terms of all drivers considered.

REFERENCES

Alto BW, Juliano SA. Precipitation and temperature effects on populations of *Aedes albopictus* (Diptera: Culicidae): implications for range expansion. J Med Entomol. 2001; 38(5): 646-56.

Campbell LP, Luther C, Moo-Llanes D, Ramsey JM, Danis-Lozano R, Peterson AT. Climate change influences on global distributions of dengue and chikungunya virus vectors. Philos Trans R Soc Lond B Biol Sci. 2015; 370(1665): pii: 20140135.

Cliff A, Haggett P. Time, travel and infection. Br Med Bull. 2004; 69: 87-99.

Dhimal M, Gautam I, Joshi HD, O'Hara RB, Ahrens B, Kuch U. Risk factors for the presence of chikungunya and dengue vectors (*Aedes aegypti* and *Aedes albopictus*), their altitudinal distribution and climatic determinants of their abundance in central Nepal. PLoS Negl Trop Dis. 2015; 9(3): e0003545.

Estallo EL, Lamfri MA, Scavuzzo CM, Almeida FF, Introini MV, Zaidenberg M, et al. Models for predicting *Aedes aegypti* larval indices based on satellite images and climatic variables. J Am Mosq Control Assoc. 2008; 24(3): 368-76.

Kalra NL, Kaul SM, Rastogi RM. Prevalence of *Aedes aegypti* and *Aedes albopictus* vectors of DF/DHF in north, northeast and central India. Dengue Bull. 1997; 21: 84-92.

Kilpatrick AM, Randolph SE. Drivers, dynamics, and control of emerging vector-borne zoonotic diseases. Lancet. 2012; 380(9857): 1946-55.

Koyadun S, Butraporn P, Kittayapong P. Ecologic and sociodemographic risk determinants for dengue transmission in urban areas in Thailand. Interdiscip Perspect Infect Dis. 2012; 907494.

Kraemer MU, Sinka ME, Duda KA, Mylne AQ, Shearer FM, Barker CM, et al. The global distribution of the arbovirus vectors *Aedes aegypti* and *Ae. albopictus*. Elife. 2015; 4: e08347.

Lobo JM, Jiménez-Valverde A, Real R. AUC: a misleading measure of the performance of predictive distribution models. Glob Ecol Biogeogr. 2008; 17(2): 145-51.

Lozano-Fuentes S, Hayden MH, Welsh-Rodriguez C, Ochoa-Martinez C, Tapia-Santos B, Kobylinski KC, et al. The dengue virus mosquito vector *Aedes aegypti* at high elevation in Mexico. Am J Trop Med Hyg. 2012; 87(5): 902-9.

Mogi M, Armbruster P, Tuno N, Campos R, Eritja R. Simple indices provide insight to climate attributes delineating the geographic range of *Aedes albopictus* (Diptera: Culicidae) prior to worldwide invasion. J Med Entomol. 2015; 52(4): 647-57.

Neerinckx SB, Peterson AT, Gulinck H, Deckers J, Leirs H. Geographic distribution and ecological niche of plague in sub-Saharan Africa. Int J Health Geogr. 2008; 7: 54.

Nelson A. Estimated travel time to the nearest city of 50,000 or more people in year 2000. Global Environment Monitoring Unit - Joint Research Centre of the European Commission, Ispra, Italy. 2008; Available from: http://forobs.jrc.ec.europa.eu/products/gam/.

Nihei N, Komagata O, Mochizuki K, Kobayashi M. Geospatial analysis of invasion of the Asian tiger mosquito *Aedes albopictus*: competition with *Aedes japonicus japonicus* in its northern limit area in Japan. Geospat Health. 2014; 8(2): 417-27.

Noor AM, Alegana VA, Gething PW, Tatem AJ, Snow RW. Using remotely sensed night-time light as a proxy for poverty in Africa. Popul Health Metr. 2008; 6: 5.

Nsoesie EO, Kraemer MU, Golding N, Pigott DM, Brady OJ, Moyes CL, et al. Global distribution and environmental suitability for chikungunya virus, 1952 to 2015. Euro Surveill. 2016; 21(20): doi: 10.2807/1560-7917.ES.2016.21.20.30234.

Owens HL, Campbell LP, Dornak LL, Saupe EE, Barve N, Soberón J, et al. Constraints on interpretation of ecological niche models by limited environmental ranges on calibration areas. Ecol Model. 2013; 263: 10-8.

Peterson A, Soberón J, Pearson R, Anderson R, Martínez-Meyer E, Nakamura M, et al. Ecological niches and geographic distributions. Princeton: Princeton University Press; 2011. 314 pp.

Peterson AT. Mapping disease transmission risk: enriching models using biogeography and ecology. Baltimore: Johns Hopkins University Press; 2014.

Phillips SJ, Anderson RP, Schapire RE. Maximum entropy modeling of species geographic distributions. Ecol Model. 2006; 190(3-4): 231-59.

R Development Core Team. A language and environment for statistical computing. R Foundation for Statistical Computing. Vienna: 2015. Available from: http://www.R-project.org.

Sena A, Barcellos C, Freitas C, Corvalan C. Managing the health impacts of drought in Brazil. Int J Environ Res Public Health. 2014; 11(10): 10737-51.

Teurlai M, Menkes CE, Cavarero V, Degallier N, Descloux E, Grangeon JP, et al. Socio-economic and climate factors associated with dengue fever spatial heterogeneity: a worked example in New Caledonia. PLoS Negl Trop Dis. 2015; 9(12): e0004211.

Vanwambeke SO, Somboon P, Harbach RE, Isenstadt M, Lambin EF, Walton C, et al. Landscape and land cover factors influence the presence of *Aedes* and *Anopheles* larvae. J Med Entomol. 2007; 44(1): 133-44.

Wang W, Cheng H, Zhang L. Poverty assessment using DMSP/OLS night-time light satellite imagery at a provincial scale in China. Adv Space Res. 2012; 49(8): 1253-64.

Weaver SC. Urbanization and geographic expansion of zoonotic arboviral diseases: mechanisms and potential strategies for prevention. Trends Microbiol. 2013; 21(8): 360-3.

Wu PC, Lay JG, Guo HR, Lin CY, Lung SC, Su HJ. Higher temperature and urbanization affect the spatial patterns of dengue fever transmission in subtropical Taiwan. Sci Total Environ. 2009; 407(7): 2224-33.
